# RECURRENT SLEEP PARALYSIS - FEAR OF SLEEPING

**DOI:** 10.1590/1984-0462/2020/38/2018226

**Published:** 2019-11-25

**Authors:** Daniela Figueiredo Ramos, Joana Magalhães, Paulo Santos, Jorge Vale, Maria Inês Santos

**Affiliations:** a Centro Hospitalar e Universitário de Coimbra, Coimbra, Portugal.; b Centro Hospitalar Tondela-Viseu, Viseu, Portugal.

**Keywords:** Sleep paralysis, Hallucinations, Anxiety, Parasomnia, Paralisia do sono, Alucinações, Ansiedade, Parassonia

## Abstract

**Objective:**

To report a case of recurrent isolated sleep paralysis (RISP), a benign parasomnia with worrisome and frightening sleep paralysis episodes.

**Case:**

**description:** We describe a case of RISP in a sixteen-year-old girl who seeks medical attention for anxiety symptoms. The sleep paralysis and associated auditory and tactile hallucinations began three years before with worsening in the last year, causing fear of sleeping. The episodes were intensely frightening causing negative impact in patient’s sleep, school performance and social function. Medical conditions were excluded, and she started treatment with a selective serotonin reuptake inhibitor with complete resolution of symptoms.

**Comments:**

Sleep complaints are often devalued. Therefore, clinicians should actively ask their patients about their sleep during health assessment.

## RESUMO

**Objetivo:**

Relatar um caso de paralisia do sono isolada e recorrente (PSIR), uma parassonia benigna com episódios inquietantes e assustadores de paralisia do sono.

**Descrição:**

**do caso:** Descreve-se um caso de PSIR de uma adolescente de dezesseis anos que buscou cuidados médicos devido a sintomas de ansiedade. A paralisia do sono e as alucinações auditivas e táteis associadas haviam começado três anos antes, com agravamento no último ano, causando medo de dormir. Os episódios eram extremamente perturbadores, gerando um impacto negativo no sono, desempenho escolar e vida social da paciente. Condições médicas foram excluídas e começou um tratamento com um inibidor seletivo da recaptação de serotonina, com resolução completa dos sintomas.

**Comentários:**

Queixas relacionadas ao sono são frequentemente subvalorizadas. Portanto, os médicos devem perguntar aos seus pacientes sobre problemas relacionados com o sono durante a avaliação clínica.

## INTRODUCTION

Sleep paralysis (SP) occurs when rapid eye movement (REM) atonia is maintained into wakefulness,^1^ ^,^ ^2^ with no other clinical features of narcolepsy^3^. Isolated SP episodes are characterized by muscle atonia with preserved ocular and respiratory movements upon sleep onset or offset,^2^ ^,^ ^3^ ^,^ ^4^ ^,^ ^5^ usually brief and which disappears spontaneously or upon external stimulation.^2^ Most individuals experience dream activity during this conscious paralysis, in a vivid, multisensorial, and often negatively valued way, making SP a very unpleasant experience.^4^ ^,^ ^6^

Isolated SP episodes are not better explained by other sleep disorders (e.g. narcolepsy), medication effects or other substances. Recurrent isolated sleep paralysis (RISP) is a benign parasomnia consisting of multiple episodes of isolated SP (at least two in six months) associated with clinically significant distress (anxiety and/or fear related to the bedroom/sleep).^1^ ^,^ ^2^

Lifetime prevalence of RISP is 7,6%,^1^ but higher prevalence has been reported among students (28,3%) and females.^1^ ^,^ ^5^ Other risk factors are poor sleep or sleep disruption, psychiatric pathology (anxiety, panic or posttraumatic stress disorder) and certain personality traits.^1^ ^,^ ^7^ In this paper, we describe a case of recurrent SP episodes associated with significant anxiety.

## CASE DESCRIPTION

A previously healthy sixteen-year-old girl was referred to the Adolescent Medicine Clinic by her Family Physician for fearfulness, inquietude, sleep disturbance and hallucinations.

She had always been anxious, usually at school, despite her good performance. At the age of nine, she was followed up by a psychologist after having witnessed her mother’s seizure due to stroke.

Three years earlier, she began having frequent episodes of total paralysis upon waking (between 6:00‒7:00 a.m.). These lasted about two minutes and were more common during holidays when she slept through the morning. She mentioned increasing frequency and duration in the last year. The episodes were also more frequent when she slept in supine position and less frequent in lateral decubitus. She also mentioned dyspnea and auditory and tactile hallucinations (“I feel a claw of an animal in my head”, “someone holding my hands”, “tight around the neck”, “friends calling my name”). Although paralysis was transient, these episodes were very frightening and led to a persistent state of anxiety and fear of sleep with decreased sleep quality, insomnia, tiredness, daytime drowsiness, poor concentration and memory with worsening in school performance, demotivation, isolation and progressive withdrawal from her peer group. There were not episodes of cataplexy or symptoms of restless legs and she denied snoring or apnea.

Despite reporting daytime somnolence, Epworth Sleepiness Scale score was 1/24. She reported laying down at 10:00 p.m. listening to music or reading on her mobile phone and falling asleep half an hour later. She woke up at 7:00 a.m. on weekdays and 8:00 a.m. during weekends and denied taking naps. She also denied alcohol and caffeine consumption, as well as use of medications or illicit substances. Her father and two paternal uncles described similar episodes, but they devalued those complaints.

Physical examination was normal, apart from her being notoriously anxious and sweaty, with adequate and cooperative discourse. Blood pressure was 113/69 mmHg (<90^th^ percentile for age, sex and height percentile); pulse was 90 beats/min and regular. Weight was 49 kg, height 152 cm (3^rd^‒15^th^ percentile) and body mass index was 22,1 kg/m^2^ (50‒85^th^ percentile) (World Health Organization child growth standards). Upper respiratory tract (with Mallampati score 1) and neck, as well as cardiopulmonary and neurologic exams, were normal.

Our differential diagnosis was: inadequate sleep hygiene, narcolepsy without cataplexy, psychiatric disease, sleep-related epilepsy, and any chronic medical condition (anemia, cardiac disease, malignancy or metabolic disorder) or acquired central nervous system disorder.

Complete blood count, metabolic panel, thyroid function, vitamin B12 and D levels, electroencephalogram and cranial computed axial tomography were all normal.

Therefore, we assumed the diagnosis of RISP and she was referred to a pneumology/sleep appointment. Actigraphy showed regular sleep schedule with mean sleep latency of 36 minutes and efficiency of 81%. Mean total sleep time was 9 hours and 10 minutes.

Despite being advised about the benign course of the disease and sleep hygiene, the complaints persisted. One month later, she was consulted by a Child and Adolescent Psychiatrist and started treatment with a selective serotonin reuptake inhibitor (oral fluvoxamine 50 mg once-daily), without adverse effects. There was a significant improvement of all symptoms, so no other diagnostic workup was made. She was under fluvoxamine for seven months with total regression of symptoms and no recurrence six months after dose tapering.

## DISCUSSION

This paper depicts a typical case of RISP. Its onset is usually in adolescence^4^ ^,^ ^6^ and more common in girls.^1^ SP episodes occur during awakening from sleep (hypnopompic) as opposed to narcolepsy-associated paralysis, which is closely associated with sleep-onset (hypnagogic).^3^ ^,^ ^4^ ^,^ ^8^ In our case, episodes lasted two minutes, while in literature an average of six minutes is described.^1^ ^,^ ^4^ Many reports indicate that events occur more often in supine position, like with this girl, although the reason is unknown.^1^ ^,^ ^3^ ^,^ ^4^ ^,^ ^5^ ^,^ ^9^ Hallucinations are usually the most disturbing symptom.^6^ ^,^ ^8^ Pressure on chest has been reported in 52,9% and feeling of being strangled in 17,6%.^4^ These are frequent (88,3%) but not essential for diagnosis.^1^

RISP diagnosis requires clinical criteria fulfillment, including frequency and clinical distress,^1^ and episodes must not be better explained by other sleep disorders, medication or substance effects.^1^ ^,^ ^4^ Many medical conditions may be associated with excessive daytime sleepiness or sleep-related symptoms,^10^ so it is essential to collect a detailed clinical history and perform adequate complementary studies. Polysomnography may be useful to support diagnosis^1^ but, in our center, given the lack of resources, it’s reserved for complex cases or cases in which patients do not respond to first-line intervention.

Patients often describe episodes as intensely frightening, even after understanding that the disorder is benign and self-limited.^3^ Fear arises from paralysis and hallucinatory experiences.^4^ ^,^ ^6^ ^,^ ^9^ Our patient developed anticipatory anxiety with great impact in her sleep quality and daily life. Patients need reassurance and also need to learn that they are not problematic or “crazy”, and should discuss these events with a health care provider to alleviate unnecessary anxiety.^1^ ^,^ ^4^ ^,^ ^6^

Experiencing traumatic events appears to be related to SP.^5^ In addition to anxiety, SP and RISP are also associated with panic and posttraumatic stress disorder.^1^ ^,^ ^6^ ^,^ ^8^ Hence, SP should be routinely assessed within certain psychiatric groups.^5^

Inadequate sleep hygiene is a risk factor for RISP.^5^ However, this patient did not present significant issues, except the use of mobile phone in bed. Nevertheless, she stated that the episodes were more frequent during the holidays, when her sleep lasted longer.

Sometimes SP carries deep cultural significance (cultural attribution of episodes to the supernatural), so patients hide such events.^2^ ^,^ ^3^ ^,^ ^5^ In this case, the episodes had been happening for at least three years before their impact became significant enough for her to seek medical help. Parents may not realize the relationship between sleep problems and daytime behavior. In this case, her mother attributed symptoms to a traumatic experience and made no remarks of her sleep disturbance.

A familial pattern is common^5^ but, like her father and uncles, some patients do not experience clinically significant distress.^1^ RISP treatment includes avoiding sleep deprivation and identifying precipitants. Tricyclic antidepressants and selective serotonin reuptake inhibitors may reduce frequency through suppression of REM sleep.^1^ ^,^ ^2^ ^,^ ^3^ ^,^ ^6^ ^,^ ^7^ Another option is cognitive behavioral therapy.^1^ Decisions on treatment are currently hampered because no randomized controlled trial has been conducted for RISP.

This is an interesting clinical case that highlights the impact of sleep disorders. The literature on this subject is scarce and, to the authors’ knowledge, no pediatric cases have been published.

This case shows that clinicians should question about sleep during routine health assessment with age-appropriate questionnaires, as sleep disorders are common and may interfere with physical, cognitive, emotional and social development.
